# One-year survey of methadone-induced cardiac complications

**DOI:** 10.22088/cjim.14.1.43

**Published:** 2023

**Authors:** Seyed Kazem Taheri, Rashed Bawand, Farid Sanginabadi, Nakisa Khansari, Abbas Moradi

**Affiliations:** 1 Department of Forensic Medicine and Toxicology, School of Medicine, Hamadan University of Medical Sciences, Hamadan, Iran; 2 School of Medicine, Hamadan University of Medical Sciences, Hamadan, Iran; 3 Department of Cardiology, School of Medicine, Hamadan University of Medical Sciences, Hamadan, Iran; 4 Department of Community Medicine, School of Medicine, Hamadan University of Medical Sciences, Hamadan, Iran

**Keywords:** Methadone, Cardiac complications, EKG, ECG, QT prolongation

## Abstract

**Background:**

Methadone is one of the most useful opioids that can be used to achieve many therapeutic goals, and also it may be abused as an illicit drug. Methadone can cause different gastrointestinal, neurological, and cardiac complications. This study was performed to obtain a better understanding of the cardiac side effects of methadone in patients with methadone poisoning.

**Methods:**

This cross-sectional study was performed on 210 samples in Sina Hospital of Hamadan in a one-year period from March 2019 to March 2020. After assessing patients who had methadone poisoning and completing their demographic information and evaluation of changes in patients' EKGs, the data was collected and analyzed by SPSS 16 software.

**Results:**

Out of 210 participants, 178 (84.8%) were males and the rest were females. The average age of the studied patients was 39.56 years old. The study found that 6.1% of methadone-poisoned patients were illiterate. It was found in this study that the most common cardiac complications of methadone intoxication were sinus tachycardia (20%), QT interval prolongation (6.64%), and sinus bradycardia (4.3%), respectively; nevertheless, 66.2% of patients did not have any EKG abnormalities.

**Conclusion:**

According to the findings, it is necessary to have continuous cardiac monitoring for patients with methadone intoxication and by transferring such findings to medical centers, steps can be taken to use methadone more intelligently.

Poisoning is one of the most common causes of emergency room visits. One of the most common types of poisoning is methadone poisoning (1). Methadone is a type of synthetic opioid and a derivative of heptamines, which is effective as an analgesic with high binding power to opioid receptors (2). Methadone can be used intravenously, orally, and subcutaneously, and is well-absorbed (2). Methadone is distributed to most tissues in the body and crosses the placenta (2). Methadone intoxication can make several kinds of complications such as gastrointestinal, urogenital, skin, cerebrospinal, cardiovascular, etc. (3). Among all of these side effects, cardiac complications due to their life-threatening potentials, have a prominent position (4, 5). For example, methadone use has been reported to cause cardiac side effects such as bradycardia, increased QT interval, and torsades de pointes arrhythmia (4, 5). In addition, Brugada-like syndrome is a complication that is reported to be associated with methadone use (5). Currently, Iran is one the countries that have the most number of opium addicts, and a large number of them are currently on methadone treatment (6). Therefore, according to the high prevalence of methadone use (and probably intoxication) in Iran and the importance of methadone-induced cardiac complications, this study was performed to identify more accurately the cardiac complications of patients with methadone intoxication.

## Methods

This study is a descriptive cross-sectional study with accessible sequential sampling with census method, from patients admitted due to a proven methadone intoxication with inclusion criteria and do not have exclusion criteria at the toxicology ward of Farshchian Hospital of Hamadan, from March 2019 to March 2020. The inclusion criteria of this study were: 1. History of recent methadone use, 2. No history of heart disease or any other underlying problems related to electrolyte disturbances, 3. No history of other drug use. The exclusion criteria of this study were: 1. Concomitant use of other opioids and other interfering drugs, 2. Having any underlying heart disease, 3. Having underlying thyroid disease, 4. Positive history of taking antiarrhythmics (such as sevoflurane, ranolazine, bepridil, sotalol, quinidine, amiodarone, ibutilide, disopyramide, f-dofetilide, dronedarone, etc.), antibiotics (such as moxifloxacin, gemifloxacin, clarithromycin, ciprofloxacin, Levofloxacin, Roxithromycin, Trimethoprim Sulfa, Erythromycin, Azithromycin, etc.), oncology drugs (such as tamoxifen, lapatinib, nilotinib, arsenic trioxide, vandetanib, etc.), anticonvulsants (such as fosphenytoin, phenytoin, felbamate, etc.), antipsychotic drugs (such as thioridazine, haloperidol, mesoridazine, chlorpromazine, etc.), and antidepressants (such as amitriptyline, imipramine, fluoxetine, desipramine, paroxetine, etc.). At the beginning of hospitalization, every single patient got appropriate intravenous fluid to get enough hydration. Before starting any kind of treatment, the vital signs of each patient including blood pressure and heart rate were controlled and recorded and after that, an initial EKG was obtained from each patient and then treatments like naloxone therapy and other necessary drugs were started. 

Firstly, a checklist containing demographic information of patients (including age, gender, level of education, and occupational situation) and dose of methadone and its method of use, was completed by the researcher without mentioning the patient`s name. Then, the main checklist containing questions about the cardiac side effects of methadone for each patient was completed (according to the initial obtained EKG). This checklist included possible cardiovascular complications that can be established by methadone intoxication including increased heart rate, QT prolongation, QT interval dispersion, the existence of pathologic U wave, Brugada-like syndrome, ST elevation, torsades de pointes, wide QRS, AF, VF, SA block, AV block, T wave inversion, ventricular bigeminy, and other cardiac complications. For the calculation of the QT interval, these two formulas were used (7):

1. QTc = QT + 1.75 (HR-60)          2. QTc = QT /$\sqrt[{3}]{\mathrm{RR}}$

The normally considered values for QT interval were (7):

Max = 450 milliseconds in men and 460 milliseconds in women Min = 390 milliseconds. Finally, at the discharging time, a secondary control EKG was obtained from patients that were discharged with the confirmation of the clinician and were compared with the primary one. All obtained information was recorded in a checklist and entered into SPSS 16 software. Findings were displayed with frequency indices (mean and standard deviation) and percentages. 

This study was approved by the Ethics Committee of Hamadan University of Medical Sciences (Ethical Code: IR.UMSHA.REC.1398.710).

## Results

**Figure 1:**
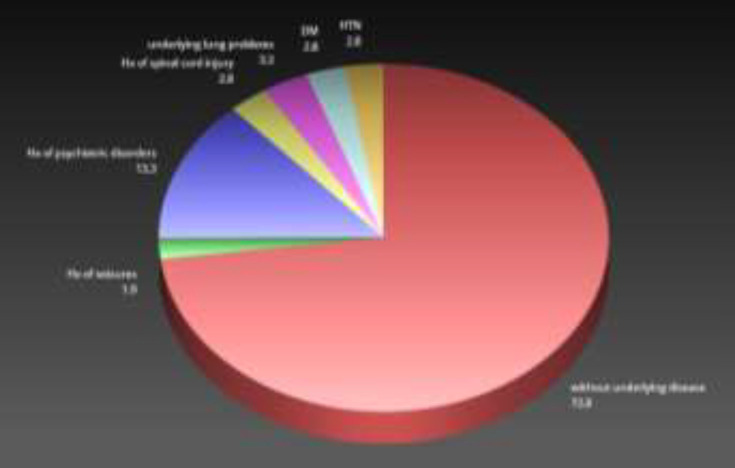
Frequency percentage of different underlying diseases among the studied population [COMMENT: The figure size needs to be increased due to not readability of its content]

After performing all inclusion and exclusion criteria, 210 patients with definite methadone toxicity diagnoses were chosen and entered the main study. In terms of demographic characteristics, the mean age of the studied population was 39.56 years old with a standard deviation of 16.41, max age of 82 years old, and min age of 14 years old. 178 (84.8%) patients were males and 32 (15.2%) were females. In terms of educational level, 13(6.1%) people were illiterate, 151(71.9%) individuals had primary educations, 10(4.7%) subjects had secondary education, 28(13.3%) individuals were high school graduates, 5(2.3%) people had post-diplomas, and 3(1.4%) had bachelor’s degree. Additionally among these participants, 107(50.9%) patients were self-employed, 15(7.1%) were disabled due to old age, 25(11.9%) patients were students, 5(2.3%) people were workers, 16(7.6%) were employees, 15(7.1%) were farmers, 22(10.4%) were housewives, and 5 patients were prisoners. In terms of the ways of using methadone, 193(91.9%) used it orally and the rest (8.1%) were unspecified. 153(72.8%) subjects were without underlying disease. 6(2.8%) with history of spinal cord injury, 7(3.3%) had underlying lung problems, 28(13.3%) with history of psychiatric disorders, 6(2.8%) had diabetes mellitus (DM), 6(2.8%) with history of hypertension (HTN), and 4(1.9%) patients had a history of seizures (Figure 1). 

The dose of methadone that caused patients to be referred to the medical center was known in only 76 of the subjects with an average dose of 331.09 mg. EKG disorders were recorded in only 71 (33.8%) subjects. In the remaining 139 (66.2%) patients, no EKG changes were seen. Different kinds of EKG disorders and their frequencies in the studied population are shown in Table 1.

At the end of treatment, 165 (78.6%) patients were discharged from the hospital with complete recovery, 20 (9.5%) patients were discharged with mild complications (such as dizziness, weakness, dry mouth, physical dependence, mild abdominal pain, etc.), 14 (6.7%) patients were discharged during treatment with personal consent, and 11 (5.2%) patients died. The control EKGs that were obtained from patients at the discharging time –from patients that were discharged with the conformation of clinician- were compared with the initial ones and approximately all the changes were reversed at almost all of the discharged patients. 

**Table 1:** Different kinds of EKG disorders and their frequencies in the studied population

Kind of EKG disorder	**Frequency**	**Percentage (%)**
**Normal EKG**	139	66.2
**Sinus tachycardia**	42	20
**Sinus bradycardia**	9	4.3
**QT prolongation**	14	6.64
**Uniform ventricular bigeminy**	1	0.48
**Pathologic U wave**	1	0.48
**Atrial Fibrillation **	2	0.95
**QT interval dispersion**	2	0.95
**Aggregation**	210	100

## Discussion

The results of this study confirmed that the most common heart complication due to methadone poisoning was tachycardia, and then QT prolongation had the second prevalence. In the study of Shakouri H et al. (8), methadone therapy was found to increase the QT interval in the electrocardiogram, which is consistent with the findings of the present study in this regard. A study by Alinejad S et al. (5), found that QT prolongation, QT scattering, torsades de pointes, ventricular bigeminy, Tako-Tsubo syndrome (stress cardiotoxicity), Brogada-like syndrome, and hemodynamic effects were among the side effects of methadone. The result of this study is consistent with the result of the present study.

In a study by Calver L et al. (9), it was concluded that methadone use, in contrast to non-use, prolongs the QT interval. The results of the above case study are also consistent with the results of the present study. In the study of Gholami et al. (3), the mean age of participants in the project was 31.71 years. The mean age in that study was lower than in the present study, which seems to be due to the larger number of samples in the present study. Also, in the study of Gholami et al. (3), 81.5% of the subjects were males. In addition, 23.2% of these people had a high school diploma or higher. Therefore, from this aspect, the results of this study are consistent with the findings of the present study.

In the study of Behdani F et al. (10), about 96% were men and 49% were employed, and about 48% of people had a diploma or higher, which is not consistent with the results of our study. Nevertheless, in terms of other demographic data, this group is almost similar to our results. In the study by Hossaini et al. (11), 155 patients with a mean age of 35 years were studied and 80% had undergraduate education. That is consistent with the demographic characteristics of the present study.

In a study by Kao et al. (12), that was about Methadone-Associated Cardiac Arrhythmia, from 1997 until 2011, 1646 cases of ventricular arrhythmia or cardiac arrest and 379 cases of QTc prolongation or torsade de pointes were found that were associated with methadone; which, shows that QTc prolongation is one of the most common side effects of methadone and the results of this study are consistent with the findings of the present study. In a study by Price et al. (13), on patients receiving methadone for pain management, no significant cardiac side effects resulting from methadone use for pain were detected. The discrepancy between the results of this study with the present study might be because of the lower dose of methadone that was used to relieve the pain compare with the high doses that caused intoxication in our study. 

In conclusion, the findings showed that out of 210 patients with methadone intoxication, 71 had ECG changes, and the most common methadone-associated cardiac complications were tachycardia and prolongation of the QT interval. In addition, the prevalence of methadone poisoning was higher in men and most poisoned patients were from the illiterate strata of society. Due to the importance of cardiac side effects, it is recommended that patients under methadone treatment, would be followed periodically from the cardiac points; and cardiac monitoring should be considered for those who present with methadone intoxication. Also, clinicians should make sure that there is no underlying heart disease, especially long QT, in patients, before prescribing methadone. Finally, given the wide range of adverse effects of methadone (including a wide range of cardiac side effects), which can be even life-threatening, it seems rational to think about some alternatives for methadone in clinical practice.
